# Influence of dust and mud on the optical, chemical, and mechanical properties of a pv protective glass

**DOI:** 10.1038/srep15833

**Published:** 2015-10-30

**Authors:** Bekir Sami. Yilbas, Haider Ali, Mazen M. Khaled, Nasser Al-Aqeeli, Numan Abu-Dheir, Kripa K. Varanasi

**Affiliations:** 1 Mechanical Engineering Department, King Fahd University of Petroleum & Minerals, Dhahran, Saudi Arabia; 2 Chemistry Department, King Fahd University of Petroleum & Minerals, Dhahran, Saudi Arabia; 3 Mechanical Engineering, Massachusetts Institute of Technology, Boston, USA

## Abstract

Recent developments in climate change have increased the frequency of dust storms in the Middle East. Dust storms significantly influence the performances of solar energy harvesting systems, particularly (photovoltaic) PV systems. The characteristics of the dust and the mud formed from this dust are examined using various analytical tools, including optical, scanning electron, and atomic force microscopies, X-ray diffraction, energy spectroscopy, and Fourier transform infrared spectroscopy. The adhesion, cohesion and frictional forces present during the removal of dry mud from the glass surface are determined using a microtribometer. Alkali and alkaline earth metal compounds in the dust dissolve in water to form a chemically active solution at the glass surface. This solution modifies the texture of the glass surface, thereby increasing the microhardness and decreasing the transmittance of the incident optical radiation. The force required to remove the dry mud from the glass surface is high due to the cohesive forces that result from the dried mud solution at the interface between the mud and the glass. The ability altering the characteristics of the glass surface could address the dust/mud-related limitations of protective surfaces and has implications for efficiency enhancements in solar energy systems.

Glass is widely used in solar energy harvesting applications to protect active devices from harsh environments, such as dust, heavy rain, wind, etc. Heavy dust storms are an environmental concern around the world, particularly in the Kingdom of Saudi Arabia. Changes in climate beyond normal expectations contribute considerably to the frequency of dust storms in the Middle East^1^, which has an adverse effect on the environment, urban life, and most industries. Air humidity and patchy rainfall cause dust accumulation to be severely problematic after dust storms. Dust accumulation occurs because of the ionic compounds in dust, which dissolve in water and modify the interfacial forces between the dust and surfaces. For dry dust particles that land on a solid surface, the interfacial forces are mainly governed by van der Waals forces. However, the interfacial forces increase in the presence of liquids, thereby resulting in mud formation on substrate surfaces. In this case, the cohesive forces due to the dried mud solution at the interface contribute to the increases in the interfacial forces.

The dust particles are composed of compounds containing alkali (NaOH) and alkaline earth metals (CaCO_3_)^1^ that dissolve in condensed water vapor and increase the pH of the water. After mud forms from dust particles and water, some of the components of the dust, such as the alkali/alkaline earth compounds, dissolve into the water and form a chemically active solution. Because mud is composed of porous structures, the solution (water with dissolved ionic compounds) forms sediments at the interface between the substrate and the mud. The solution dries and forms a crystalline layer between the dried mud and the substrate. This process modifies the surface chemistry (i.e., the adhesive and cohesive forces) and increases the force required to remove the mud from the surface because of the additional covalent bonding. Because the mud formation and removal are interrelated, these processes are complex and require a thorough investigation of the after-effects of mud deposition, including the chemical, optical, morphological, and mechanical (adhesion, friction, hardness, etc.) effects on the substrate surfaces. Consequently, investigation of the mud formed from dust on glass surfaces and its after-effects on the surface characteristics is essential.

A considerable number of research studies have been performed to examine the effects of dust on various solid surfaces. Dust accumulation on an insulator and the adhesion force between dust particles and the insulator surface were studied by Wang *et al.*^2^. They found that the charging of an insulator surface produces long-range attractive forces on the dust particles; however, such charging had little influence on the adhesion force. The effects of sand and dust accumulation on photovoltaic modules were studied by Beattie *et al.*^3^. They demonstrated that the reduction in the active area of the modules was mainly due to the formation of clusters of particles on the surface, which reduced the available area for light capture to a much smaller area compared to particles resting directly on the glass surface. The adhesion of dust particles to common indoor surfaces in an air-conditioned environment was examined by Tan *et al.*^4^. They found that dust and activated carbon adhesion were highly sensitive to surface roughness with an inverse relationship between adhesion force and roughness due to the reduction in contact area between the particle and a rougher material surface. The effect of drought on dust production in the Sudano–Sahelian zone of the Sahara Desert was investigated by Middleton^5^. The data showed that dust-storm activity in the west and east of the Sudano–Sahelian belt had dramatically increased during the drought years; by a factor of 6 in Mauritania and up to a factor of 5 in Sudan. The transport of Asian dust around the globe was reported by Uno *et al.*^6^. The findings revealed that Asian dust could influence the global radiation budget by stimulating cirrus cloud formation and marine ecosystems by supplying nutrients to the open ocean. The eolian dust deposition in the western United States due to human activity was studied by Neff *et al.*^7^. They indicated that the larger dust flux, which persisted into the early twenty-first century, resulted in more than fivefold increase in inputs of K, Mg, Ca, N and P to the alpine ecosystems, with implications for surface-water alkalinity, aquatic productivity and terrestrial nutrient cycling.

A study of the impact of airborne dust deposition on the performance of solar photovoltaic (PV) modules was performed by Jiang *et al.*^8^. They demonstrated that dust pollution had a significant impact on PV module output and that the reduction of efficiency had a linear relationship with the dust deposition density; however, no differences due to the cell type were noted. The effectiveness of self-cleaning and antireflective packaging glass for solar modules was examined by Verma *et al.*^9^. They indicated that non-lithographic nanostructuring of the packaging glass surface resulted in both reduced reflection at the air/glass interface and self-cleaning characteristics. The effects of dust on the transparent covers of solar collectors were studied by Elminir *et al.*^10^. The findings revealed that the reduction in glass normal transmittance depended strongly on the dust deposition density in conjunction with the plate tilt angle and the orientation of the surface with respect to the dominant wind direction. The electrodynamic screen performance for dust removal from solar panels and solar hydrogen generators was investigated by Mazumder *et al.*^11^. When the electrodes were activated by phased voltage, the dust particles on the surface of the film became electrostatically charged and were removed by the traveling wave generated by the applied electric field.

The influence of the dust deposition on the performance of parabolic trough solar collectors was studied by Niknia *et al.*^12^. They developed a new correlation for the thermal performance of a parabolic collector due to various dust thicknesses in comparison to a clean collector. The outdoor performance and durability testing of anti-reflecting and self-cleaning glass for photovoltaic applications was presented by Sakhuja *et al.*^13^. The nanostructured glass samples resulted in enhanced self-cleaning and improved PV performance over a long exposure period. The effect of dust accumulation on the performance of evacuated tube collectors was examined by El-Nashar^14^. He reported the performance decline of evacuated tube collectors due to dust accumulation over periods extending from one month to an entire year. The energy yield loss caused by dust deposition on photovoltaic panels was investigated by Sayyah *et al.*^15^. They provided a database for predicting anticipated soiling losses at different locations around the world, which could be used to assess effective cleaning methods for restoring a system’s energy yield.

Suppression of dust adhesion on a photovoltaic concentrator module using an anti-soiling photocatalytic coating was studied by Sueto *et al.*^16^. Their findings revealed that the presence of electrostatic charges on the surfaces of the samples was a main factor in the adhesion of sand, which could be suppressed by the anti-soiling photocatalytic layer. The importance of cleaning concentrated photovoltaic arrays in a desert environment was presented by Khonkar *et al.*^17^. They indicated that in a desert environment, some extreme weather events occur that require cleaning of all types of PV modules for improved system performance. The effect of soiling on photovoltaic modules was investigated by Appels *et al.*^18^. They demonstrated that special coatings on glass had potential for reducing the power loss caused by dust settlement; however, the extra cost associated with these coatings was not justified for photovoltaic applications.

Although many research studies have been performed on dust accumulation on PV and solar thermal surfaces^15^,^16^,^17^,^18^, the chemo-mechanical effects of dust accumulation on glass surfaces in humid environments requires further investigation. Therefore, in the present study, the dust characteristics and effects of dust accumulation and mud formation (reflecting the influence of the humid air environments) on plain glass surfaces are investigated. The chemical effects of mud on glass surfaces are assessed using various analytical tools, including optical, electron scanning and atomic force microscopies, X-ray diffraction, energy dispersive spectroscopy, and Fourier transform infrared spectroscopy. The adhesion force between mud and glass surfaces is obtained from microtribometer data, and the optical transmittance of the glass after the mud removal is measured. It should be noted that the work presented is original and has not been reported elsewhere.

## Experimental

PV protective glass samples with dimensions of 30 mm × 30 mm × 2 mm (width × length × thickness) were used as workpieces. The chemical composition of the glass was 76.5% SiO_2_, 9.9% CaO, 1.2 MgO and 12.4% Na_2_O. The dust was collected from PV modules in the area of Dhahran in Saudi Arabia after a dust storm in 2014. Characterization of the dust was performed using SEM, EDS, and XRD. A JEOL 6460 scanning electron microscope was used for the SEM and EDS examinations, and a Bruker D8 Advanced diffractometer with a Cu-K_α_ radiation source was used for XRD analysis. The typical settings of the XRD instrument were as follows: 40 kV and 30 mA for the x-ray source and a scanning angle (2θ) range of 20°–80°. Roughness measurements and surface profile characterization were performed using a 5100 AFM/SPM Microscope by Agilent in contact mode. The probe tip was made of silicon nitride (r = 20–60 nm) with a manufacturer specified force constant, k, of 0.12 N/m.

A Micro Photonics digital microhardness tester (MP-100TC) was used for the surface microhardness measurements. The standard test method for the Vickers indentation hardness of advanced ceramics (ASTM C1327-99) was adopted. The measurements were repeated five times at each location to ensure the consistency of the results. A linear microscratch tester (MCTX-S/N: 01-04300) was used to determine the friction coefficient of the glass surfaces. The contact load was set at 0.03 N, and the end load was set at 2.5 N. The scanning speed was 5 mm/min, and the loading rate was 0.01 N/s. The total length for the scratch tests was 0.5 mm.

The optical transmittance was measured using a UV spectrometer (Jenway—67 Series spectrophotometer), and Fourier transform infrared spectroscopy (Bruker – VERTEX70) was performed to collect the infrared absorption spectrum of the glass.

To investigate the effects of dust and mud on the surface characteristics of the glass, actual dust accumulation and mud formation were simulated in a laboratory. In actual environments, the mud formed from accumulated dust particles due to the condensation of water vapor onto the particles. The accumulated dust thickness was measured over the period of two weeks during a dust storm in Saudi Arabia in 2014. This accumulation was on the order of 300 μm. To simulate dust accumulation in the laboratory, 300-μm layers composed of dust particles collected from the local environment were formed on the cleaned glass surfaces. Desalinated water, which was equal to the amount of water vapor that condensed on the same volume of the dust in the open environment, was dispensed gradually onto the dust layer. The initial condensation tests were performed in ambient humid air to estimate the amount of condensate that accumulated over time. Moreover, the dispensed water was left on the surface of the dust layer without mechanical mixing to resemble water condensation from humid air. Therefore, the simulated formation of mud on the glass surfaces was similar to the deposition that occurred naturally in the open environment. Next, the glasses were kept in ambient air at room temperature for three days to dry. Scratch tests were performed to measure the tangential force required to remove the mud from the glass surfaces. The tangential force provided information regarding the adhesion, cohesion and frictional work during the dry mud removal. To examine the after-effects of the mud on the glass surfaces, the dry mud was removed from the workpiece surfaces using a desalinated water jet that was 2 mm in diameter with a velocity of 2 m/s. The cleaning process was applied for 15 minutes to each glass surface. Finally, the morphology, optical transmittance, molecular characteristics, and microhardness of the mud removed glass surfaces were analyzed using the analytical tools.

The microhardness, friction and adhesion tests were repeated 12 times to secure the confidence levels for the experimental uncertainty assessments. Based on the distribution of the experimental data, the confidence level of 95% was resulted; in which case, the mean (μ) of the data distribution was within ± 1.75 of the standard deviation of the distribution of a single measurement from that distribution. The experimental uncertainty analysis revealed that the uncertainty less than 2% was resulted for the microhardness measurements while the uncertainty of about 3% was obtained for the friction and adhesion tests.

## Results and Discussion

Characterization of the environmental dust is presented, and the influence of the mud formed from the dust particles in the humid environment on the chemical and optical characteristics of the glass is examined. The frictional, adhesion, and cohesion forces required to remove the dry mud from the glass surface are also measured.

**Figure 1:**
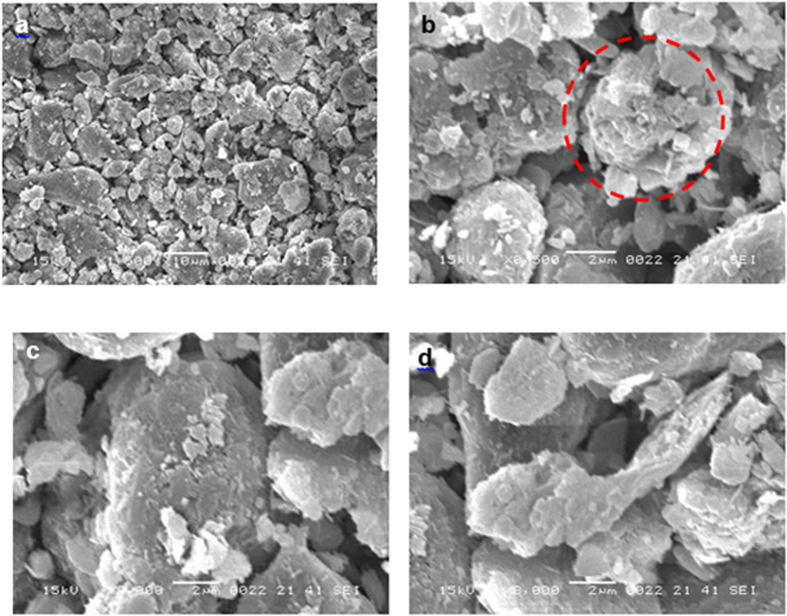
SEM micrographs of the dust particles: (**a**) dust particles of various sizes, (**b**) aggregated small particles (highlighted by the circle), (**c**) small particles attached to large particles, and (**d**) flake-like particle.

**Figure 2:**
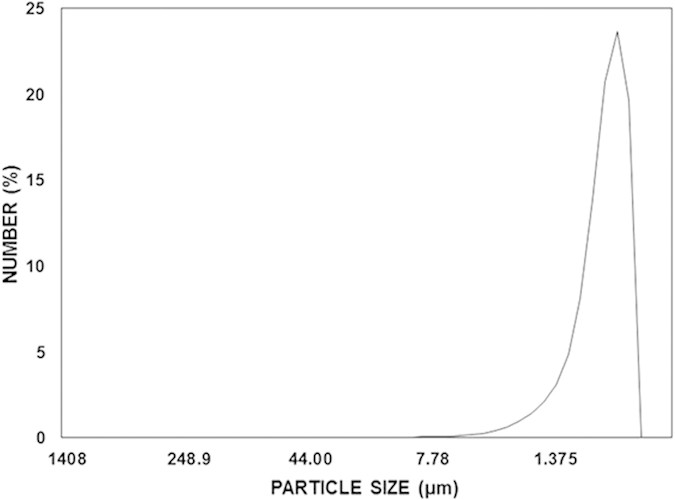
Size distribution of the dust particles.

Figure 1 shows SEM micrographs of dust particles with various sizes, and Fig. 2 shows the size distribution of the dust particles. The dust collected on the glass surfaces consists of large and fine particles and is a heterogeneous mixture of particles with various morphologies. In this case, no standard set of sizes is dominant in the dust particles mixture. However, in general, small dust particles are aggregated in clusters of 1–3 μm (Fig. 1). The large particles are on the order of 20 μm in diameter, and some small particles are attached to the surfaces of the large particles because of the charges. Although the small particles that are attached to the large particles vary in size, in general, these particles are sub-micron in size (Fig. 1). The slight bright appearance of the small particles in the SEM micrographs is associated with electron charging during the imaging. This charging indicates that these particles possess charges that allow them to attach to the large particles. As indicated in an earlier study^19^, the smaller particles (average particle diameter ≤2.5 μm) reside in the atmosphere for prolonged times and interact with solar radiation for longer durations than the large particles. Therefore, prolonged exposure to the atmosphere in regions closer to the sea causes the attachment of ionic compounds. Moreover, although the particles have irregular shapes, the average dust particle size based on the particle distribution is on the order of 1.2 μm (Fig. 2).

By analyzing the SEM images of the dust particles, two principle quantities were identified: the aspect ratio and the shape factor. This result is in agreement with the previous study^20^. The aspect ratio is 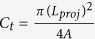, where *A* is the cross-sectional area, and *L*_*proj*_ is the longest projection length of the dust^20^. The shape factor is 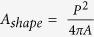, where *P* is the perimeter of the dust particle^20^. The particle diameter and area can be generated from these measurements. The diameter of a circle with equivalent area is considered for circular dusts, and for non-circular dusts, an ellipse model is used by assuming the longest projection as the major axis and preserving the cross-sectional area of the particle^20^. The aspect ratio is related to the particle roundness and approximately represents the ratio of the major axis to the minor axis of the ellipsoid best fit to the particle. In addition, the shape factor is the inverse of the particle circularity, which is associated with the complexity of the particle. In this case, a shape factor of unity corresponds to a perfect circle. The aspect ratio and the shape factor are found to change with the particle size. Although no linear relation holds between the aspect ratio or the shape factor and the particle size, the particle aspect radio decreases with increasing particle size, while the shape factor increases with increasing particle size. Nevertheless, the shape factor approaches unity for the smaller particles, while for the large particles, the median shape factor almost reaches 3.

**Figure 3:**
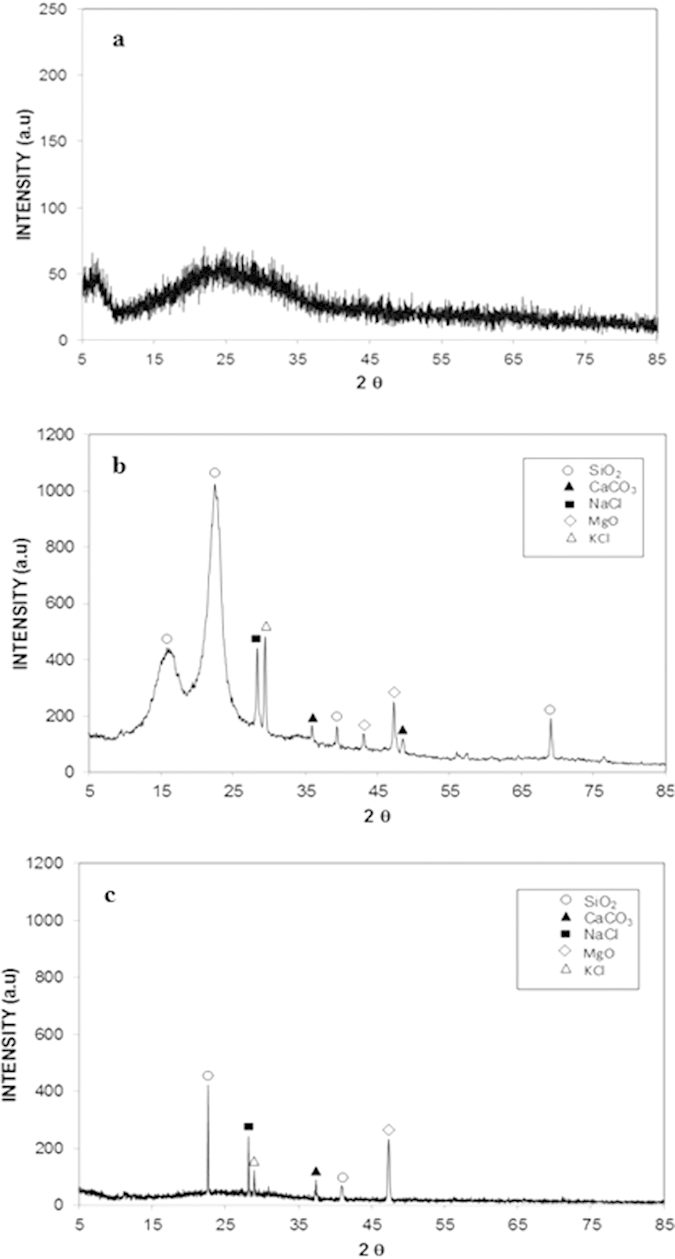
X-ray diffractograms of the different types of glass: (**a**) as-received glass, (**b**) dry mud on the glass surface, and (**c**) after removal of the dry mud from the glass surface.

Table 1 presents the EDS data for the dust particles. The presence of oxygen, iron, sulfur, chlorine, calcium, silicon, sodium, magnesium, and potassium are evident, and their concentrations vary at different locations in the dust. This result indicates that the dust is composed of non-uniformly distributed elements and compounds. The quadrangular particle, which appears to be deformed from a cubic particle, is rich in sodium and chlorine; however, the aggregated particles are rich in calcium and oxygen. Flake-like particles are also observed; these particles are rich in calcium and silicon (Fig. 1). Figure 3 shows an X-ray diffractogram of the dust particles. Potassium, sodium, calcium, sulfur, chlorine, and iron peaks are clearly visible. The iron peak is coincident with the aluminum and silicon peaks. The presence of sodium and potassium peaks is associated with sea salt because the region where the dust was collected is close to the Arabian Gulf. The concentration of chlorine changes for different dust particles, and the EDS data do not satisfy the molar ratio for NaCl (Table 1); therefore, the dust particles do not contain salt crystals; instead, NaCl is dissolved in the compound form. The sulfur may form a monomer layer during the aging process in the atmosphere. However, the sulfur can be correlated with the calcium in the dust, such as the anhydrite or gypsum component (CaSO_4_). The iron is most likely related to clay-aggregated hematite (Fe_2_O_3_).

**Figure 4:**
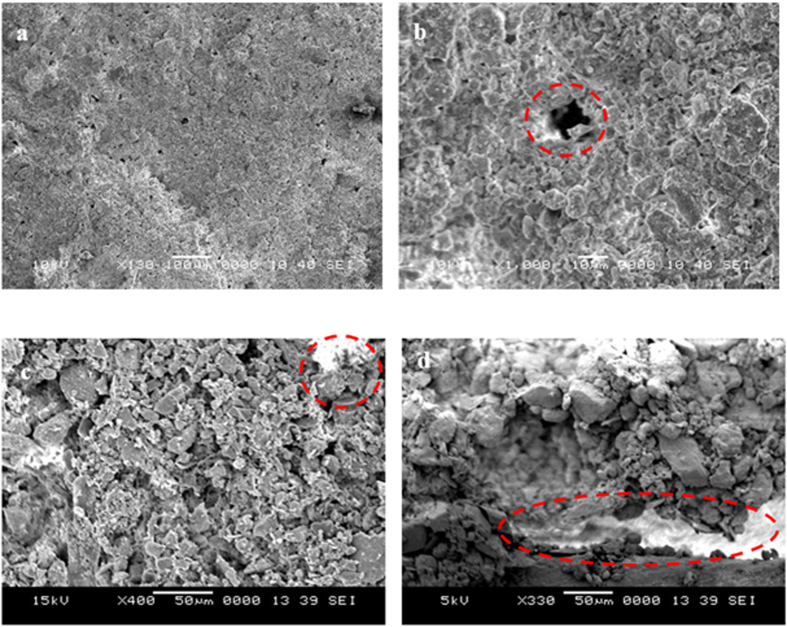
SEM micrographs of the dry mud on the glass surface: (**a**) top view of the mud at the glass surface, (**b**) cavity formed due to agglomeration of large particles in the mud (highlighted by the circle), (**c**) dry mud cross-section (white color represents the dried solution, as indicated by the circle), and (**d**) dry mud glass interface (white color is dry mud solution at the interface, as highlighted by the ellipse).

Figure 4 shows SEM micrographs of the top surface and a cross section of the mud formed on the glass surface. The mud is formed using the collected dust and the desalinated water application that mimics the condensation of water from humid air. During the formation of the mud, no mechanical mixing is used, and the mud was left to dry at room temperature for two days prior to analysis. The mud surface consists of closely adhered fine dust particles and cavities in-between the large particles (Fig. 4). Closely packed particles result in cohesive forces in the mud while improving the microhardness in this region (Table 2). Because no strong bonding occurs in-between the large particles because of the cavities formed in-between them, the microhardness becomes small in this region. Note that the microhardness of the individual large dust particles (within the range of 20 μm) is on the order of 26.3 HV. Close examination of the surface reveals the presence of a residue from the dried liquid solution in-between the small particles. The alkali and alkaline earth metals (Na and Ca) in the particles dissolve in the water during the mud formation, forming a liquid mud solution. Therefore, when the liquid solution dries, some of the dissolved alkali and alkaline earth metal compounds remain in-between the fine-sized dust particles. These regions appear as a bright color in the SEM micrographs. The presence of alkali metals is also evident from the EDS data obtained from the top surface of the dry mud (Table 3). The presence of undissolved large dust particles results in the formation of pores throughout the dry mud. The pores appear to be randomly scattered throughout the mud cross-section. The water containing dissolved mud compounds flows in-between the large particles through the cavities and eventually reaches the surface of the glass due to the effects of gravity. However, some of the mud solution is retained in the cavities in the mud forming a dense structure with a white color in the cavity upon drying. The EDS analysis reveals that the dried mud solution in the cavities exhibits similar characteristics to that at the interface between the glass and the mud. Chlorine, sodium, potassium, and sulfur are observed in the EDS data (Table 3). The dust and water mixture is ultrasonically shaken in a tube for one hour, and then, the solution is extracted; the pH of the mud solution is 8.4, which increases the concentration of OH^−^ ions in the solution. As the solution dries, it forms crystal structures at the interface and in the cavities, which appear white (Fig. 4).

**Figure 5:**
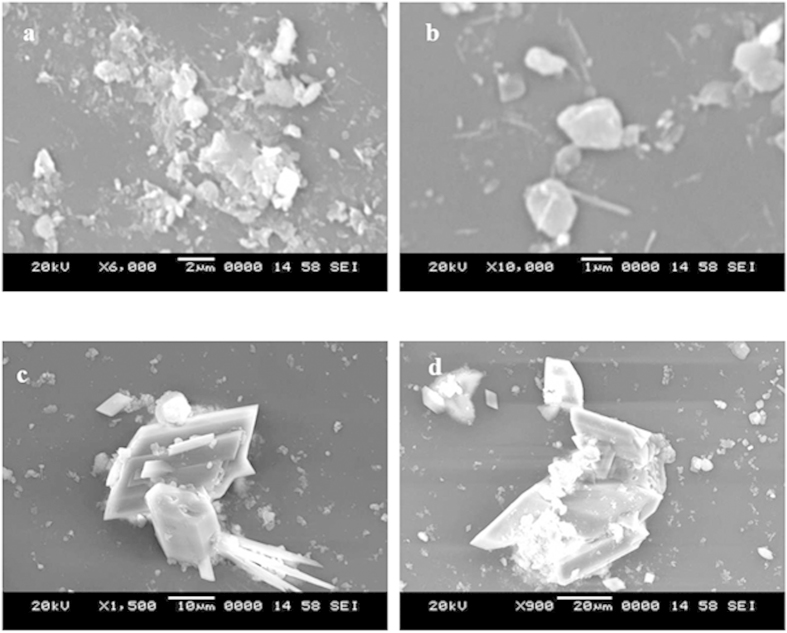
SEM micrographs of mud residues after mud removal and the dried solution: (a) large mud residue strongly attached to the glass surface, (b) fine-sized mud residue, (c) and (d) crystals formed due to the dried solution at the glass surface.

**Figure 6:**
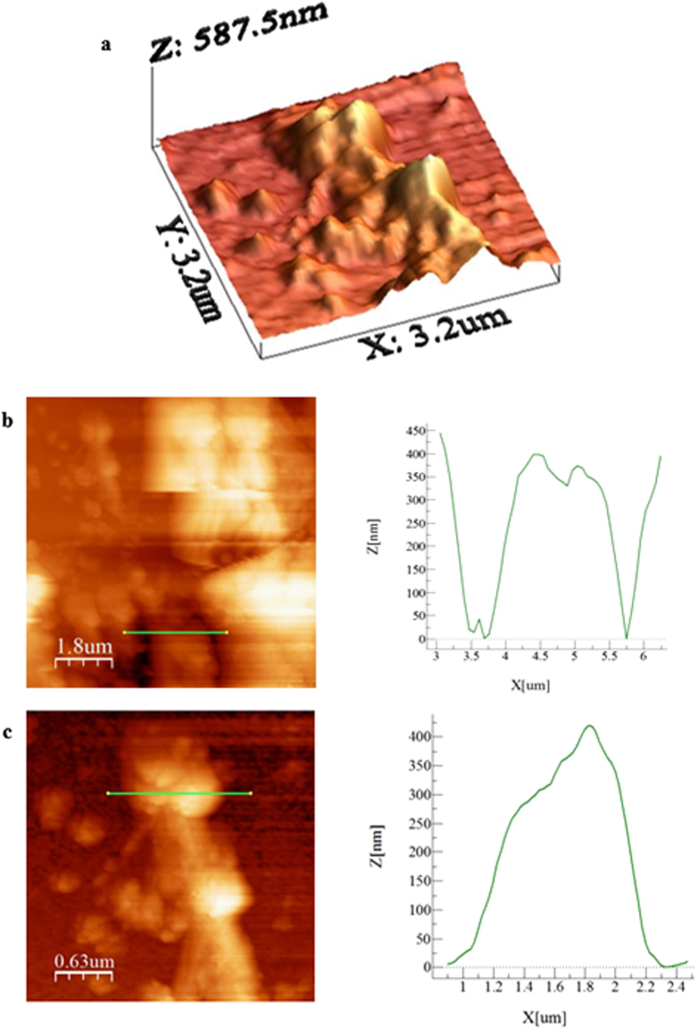
Atomic force microscopy micrographs: (a) fine-sized mud residue attached to the glass surface, (b) small cavities formed on the glass surface, and (c) horizontal profile of the mud residue.

**Figure 7:**
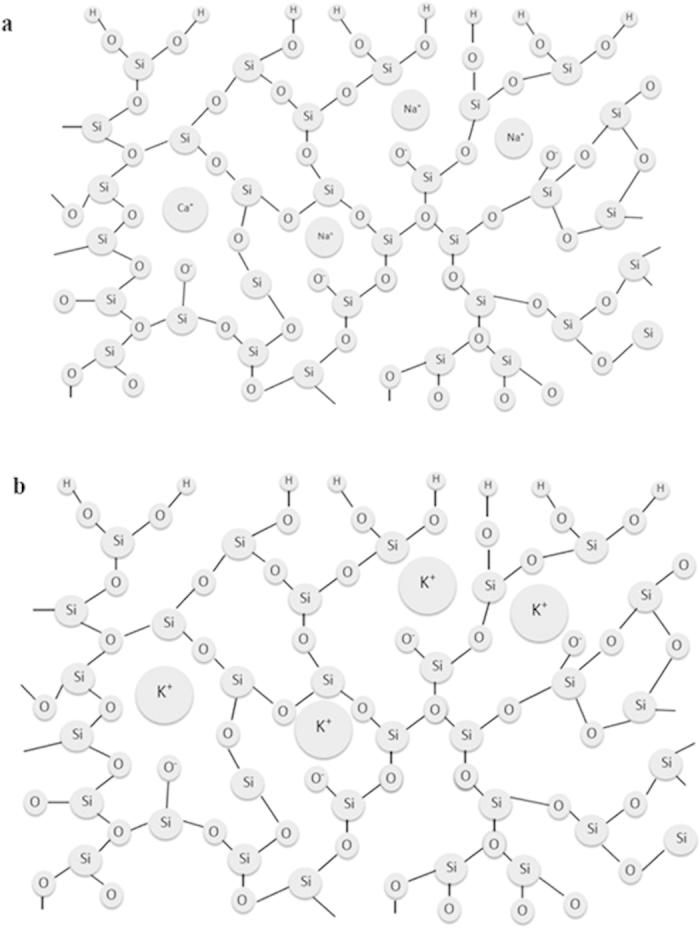
Schematic view of potassium diffusion in glass: (a) the glass structure and (b) potassium diffusion in the glass structure.

To assess the morphology and the effects of the dried mud solution at the interface, the dried mud is removed from the glass surface using a pressurized desalinated water jet with a diameter of 2 mm and a jet velocity of 2 m/s. Figure 5 shows SEM micrographs of the glass surface after the mud removal. Mud residues of different sizes are visible in the micrographs (Fig. 5). Close examination of the glass surface reveals that fine-sized crystals are formed on the surface (Fig. 5). These particles are associated with the residue from the mud solution after the removal of the dry mud. This situation is observed in the atomic force microscope images shown in Fig. 6. Moreover, due to the presence of OH^−^ ions in the mud solution (pH = 8.4), small cavities are formed in the glass surface because of surface etching. In this case, KOH ions in the mud solution are responsible for the local etching of the glass surface. The local etching increases the surface texture of the glass after the mud removal, as indicated by the line profile shown in Fig. 6. The possible ion exchange mechanism of the glass-alkaline ions at the interface between the glass surface and the mud in the presence of the mud solution can be expressed as^21^ Si − O^−^ … K^+^(OH)^−^ water → ≡ Si − OH + K^+^ + OH^−^. The breakdown of the glass network through alkaline attack due to OH^−^ ions also generates the reaction Si − O − Si ≡ + OH^−^ ≡ Si − OH + ^−^O − Si ≡. Note that KOH attack causes the formation of silanol groups (≡ Si − OH) on the glass surface, which produces cracks in the glass. Moreover, the OH^−^ ions destroy siloxane bonds (≡ Si − O − Si ≡) at the glass surface during the alkaline attack, which favors the penetration of water molecules (H^+^ and OH^−^ ions) into the glass. This process results in the formation of small cavities in the surface of the glass (Fig. 6). During the KOH attack, the diffusion of potassium ions into the surface causes a volume change because of the large diameter of potassium. This volume change results in chemical toughening of the glass surface and an increase in the microhardness of the surface after the mud removal (Table 2). Figure 7 shows a schematic view of the potassium diffusion into the glass.

**Figure 8:**
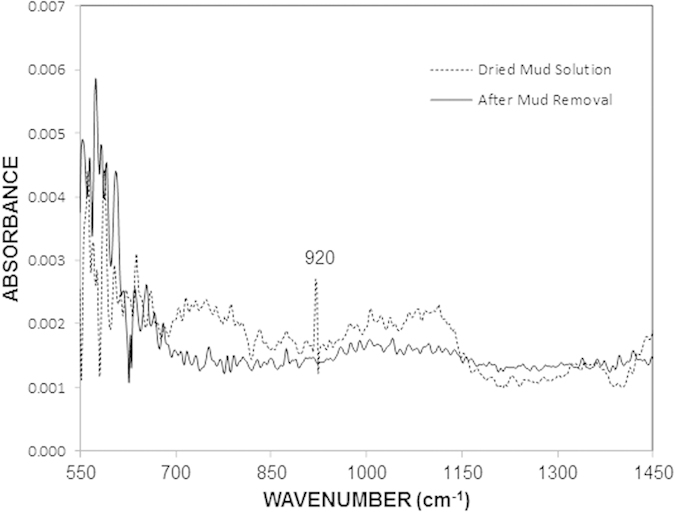
FTIR data for the as-received glass and the glass after dust removal.

**Figure 9:**
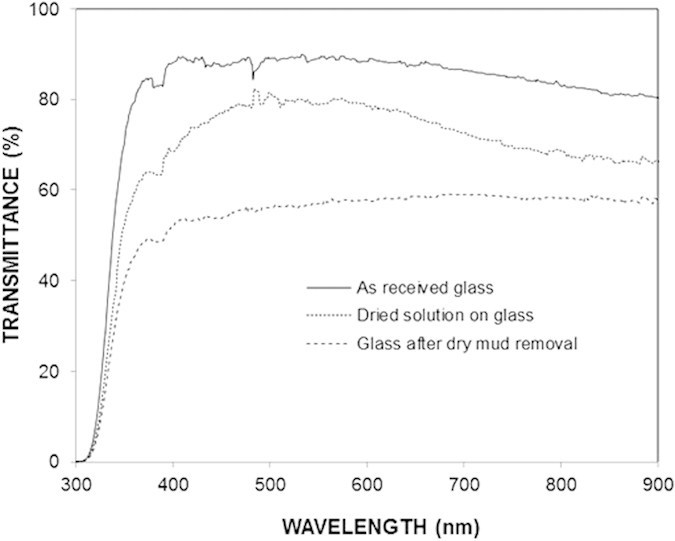
Transmittance of the as-received glass, glass after dry mud removal, and glass with the dried solution.

To examine the effects of the dry mud solution on the molecular characteristics of the glass, Fourier transform infrared (FTIR) spectroscopy was performed on the as-received glass and the glass after the removal of the dry mud. Figure 8 shows the FTIR data for the as-received glass and the glass after mud removal. In the case of the as-received surface, the 550 to 600 cm^−1^ region corresponds to the Si – O – Si bending vibration^22^, and the stretching vibration of Si – O – Si occurs at approximately 910 and 990 cm^−1^ 22. In the case of the glass surface after the mud removal, the absorbance peaks exhibit a slightly different behavior than those of the as-received glass. This difference is due to the stress induced in the region close to the glass surface due to the attack by the alkali and alkaline earth hydroxides and the diffusion of potassium into this region^22^. The stretching vibration of non-bridging oxygens (Si – O^−^) is observed at 920 cm^−1^. This peak appears in the glass after dust removal, but it is not observed in the as-received glass. The peak is related to the ionic bonding between the non-bridging oxygen and the network modifiers. In this case, the dissolution of the glass network and the formation of a new SiO_2_-enriched network due to surface reconstruction are responsible for the stretching vibration of the non-bridging oxygen^23^. Figure 9 shows the data obtained from the transmittance measurements for the as-received and the mud-removed glasses. The transmittance is reduced by nearly 35% (on average) for the mud-removed glass surface. The reduction in the transmittance is associated with (a) the mud residues, which block the incident radiation, and (b) the molecular changes that occur in the surface region of the glass due to the alkali and alkaline earth hydroxide attack.

The mud residues form strong bonds on the glass surface, which prevents the pressurized desalinated water jet from removing them (Fig. 6). The total area covered by the mud residue is estimated to be 3%. This result indicates the strong adhesion of the mud residues remaining on the glass surface. The X-ray diffractogram of the dust residues on the glass surface after the dry mud removal reveals CaCO_3_, NaCl, and MgO peaks (Fig. 3). Similar peaks are also observed in the X-ray diffractogram of the dry mud surface (Fig. 3). Because the adhesion of the dry mud to the glass surface is strong and mud residues remain after the surface is cleaned with a jet of desalinated water, the adhesion, cohesion, and frictional work required to remove the dry mud from the glass surface were determined from the tangential force analysis. Several forces can contribute to the adhesion of the mud to the glass surface, including van der Waals and electrostatic forces^24^. In the case of the dry mud on the glass surface, the dried solution, which is composed of dissolved alkali metals, alkaline earth metals, and other ions, forms a fine layer at the interface between the dry mud and the glass surface. Consequently, considering only strong van der Waals forces may not correctly describe the adhesion force because of the covalent bonds formed by the dried solution at the interface.

**Figure 10:**
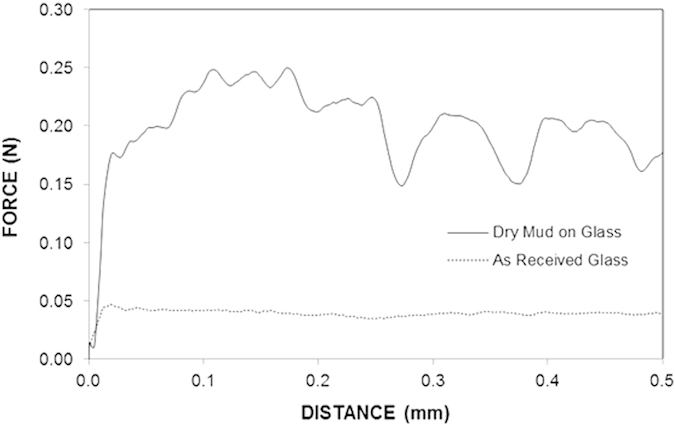
Frictional force for the as-received glass surface and the tangential force required to remove the mud from the surface.

The tangential force measured by the microtribometer when removing the dry mud from the glass surface is the combination of the adhesive and cohesive forces. The tangential force due to the as-received glass surface is associated with the frictional force; however, the tangential force due to the mud on the glass surface includes the frictional, adhesion, and cohesive forces. Therefore, integration of the tangential force over the distance traveled by the microtribometer gives the work done against friction for the as-received glass surface, while it provides the frictional, cohesive and adhesion work for the dry mud on the glass surface. The cohesion and adhesion work can be obtained after subtracting the frictional work from the work performed due to friction, cohesion, and adhesion. Figure 10 shows the tangential force variation with distance for (i) the as-received glass and (ii) the dry mud on the glass surface. Table 4 presents the work performed against friction and the combination of the cohesion and adhesion work. Table 4 shows that the frictional work is considerably smaller than the work corresponding to the combination of cohesion and adhesion. In addition, the coefficient of friction for the mud removed glass surface is larger than that of the as-received glass surface. This difference is associated with the mud residues remaining on the surface and the fine-sized cavities formed due to the KOH attack. The tangential force measurements are repeated five times, and the experimental error is estimated to be approximately 6%.

## Conclusion

The characteristics of environmental dust particles and the mud formed from these dust particles were studied. The properties of glass after the formation mud on its surface were analyzed. The local humid air conditions were simulated to enable the formation of mud on glass. Various analytical tools, including optical, scanning electron, and atomic force microscopies, X-ray diffraction, and energy dispersive spectroscopy, were used to analyze the dust and mud characteristics. The adhesion and cohesion work related to the removal of dry mud from glass were obtained using a microtribometer. The effects of the mud solution on the chemistry of the glass surface were also examined. The main findings show that the dust particles were found to be composed of a non-uniform distribution of alkali and alkaline earth metals, oxygen, silicon, sulfur, iron, etc. The average size of the particles is on the order of 1.2 μm. Morphological examination of the dust particles revealed that the dust shape factor approaches unity for the smaller particles, while for the large particles, the median shape factor reaches almost 3. The small dust particles (≤0.5 μm) were attached to the surfaces of the large dust particles (10 ≤ 20 μm) due to the presence of electrostatic charges. The mud formed from the dust on the glass surface significantly influences the glass properties, including the absorption, transmittance, microhardness, and surface texture characteristics. The dissolution of the alkali and alkaline earth compounds in the mud forms a chemically active solution, and the active solution accumulates at the interface between the mud and the glass due to gravity. Because the mud solution contains alkali and alkaline earth hydroxides, it has a pH that reaches approximately 8.4 (basic). The mud solution attacks the glass surface while altering the surface texture and the molecular vibrational states in the surface region. In addition, the diffusion of potassium into the surface region causes toughening of the surface, while increasing the surface microhardness of the glass. The optical transmittance of the glass decreases after the removal of the mud; this reduction is associated with (i) mud residues that remain after cleaning the glass surface and (ii) chemical changes in the glass surface due to the alkali and alkaline earth hydroxide attacks. The adhesion and cohesion work required to remove the mud from the glass is higher than the frictional work performed against the glass surface.

## Additional Information

**How to cite this article**: Yilbas, B. S. *et al.* Influence of dust and mud on the optical, chemical, and mechanical properties of a pv protective glass. *Sci. Rep.* **5**, 15833; doi: 10.1038/srep15833 (2015).

## Figures and Tables

**Table 1:** Elemental composition of the dust (wt%).

Si	Ca	Na	S	Mg	K	Fe	Cl	O
12.3	8.2	3.6	2.4	2.6	1.2	1.1	0.9	Balance

**Table 2:** Microhardnesses of the as-received glass, dust, dry mud, and glass surface after mud removal.

	Hardness (HV)
As-Received Glass Surface	175 (+10/−10)
Dust Particle	26.3 (+1/−1)
Dry Mud Surface	20.2 (+1/−1)
Glass Surface after Mud Removal	210 (+10/−10)

**Table 3:** EDS data from the surface of the mud, the glass surface after mud removal, and the dried solution at the interface.

	Si	Ca	Na	S	Mg	K	Fe	Cl	O
Dried Mud Surface	16.3	7.2	0.4	2.2	1.7	0.2	0.4	0.1	Balance
Mud Removed Glass Surface	11.7	6.3	3.8	1.4	2.7	1.1	0.5	—	Balance
Dried Solution at the Interface	—	7.1	2.1	1.1	1.8	1.2	0.4	0.6	Balance

**Table 4:** Frictional and adhesion work obtained from the tangential force.

	**Frictional Work (mJ)**	**Adhesion and Cohesion Work (mJ)**
As-Received Surface	0.000403	—
As-Received Surface with Mud	—	0.9213
Surface with Mud Residue	0.0293	—
